# The Multifaceted Histidine-Based Carriers for Nucleic Acid Delivery: Advances and Challenges

**DOI:** 10.3390/pharmaceutics12080774

**Published:** 2020-08-14

**Authors:** Jiaxi He, Songhui Xu, A. James Mixson

**Affiliations:** Department of Pathology, University Maryland School of Medicine, 10 S. Pine St., University of Maryland, Baltimore, MD 21201, USA; jiaxi.he@som.umaryland.edu (J.H.); songhuixu@som.umaryland.edu (S.X.)

**Keywords:** polymers, histidine, imidazole, peptides, nucleic acids, nanoparticles, polyplexes, gene therapy

## Abstract

Histidines incorporated into carriers of nucleic acids may enhance the extracellular stability of the nanoparticle, yet aid in the intracellular disruption of the nanoparticle, enabling the release of the nucleic acid. Moreover, protonation of histidines in the endosomes may result in endosomal swelling with subsequent lysis. These properties of histidine are based on its five-member imidazole ring in which the two nitrogen atoms may form hydrogen bonds or act as a base in acidic environments. A wide variety of carriers have integrated histidines or histidine-rich domains, which include peptides, polyethylenimine, polysaccharides, platform delivery systems, viral phages, mesoporous silica particles, and liposomes. Histidine-rich carriers have played key roles in our understanding of the stability of nanocarriers and the escape of the nucleic acids from endosomes. These carriers show great promise and offer marked potential in delivering plasmids, siRNA, and mRNA to their intracellular targets.

## 1. Introduction

Of the 22 proteinogenic amino acids, the biochemical properties of histidine arguably demonstrate the most diversity. These characteristics include pH-buffering, hydrogen bonding, aromaticity, coordination bonds with transitional metals, and alkylation of the ring, which modifies the hydrophobicity of the imidazole ring. Various biological systems have utilized these properties of histidine and its imidazole ring to sustain life such as enzymatic activity [1], transcriptional factors [2], the release of oxygen from hemoglobin [3], and antimicrobial activity [4]. Investigators have adopted these properties of histidines/imidazole to develop improved carriers of nucleic acids. To advance nucleic acid delivery, imidazoles/histidines incorporated into carriers have a range of roles, such as enhancing extracellular stability of polyplexes, increasing disruption of polyplexes within acidic endosomes, and augmenting endosomal lysis by osmotic swelling. Histidine-containing carriers have demonstrated significant potential to import into the cytosol different forms of nucleic acids.

**Figure 1:**
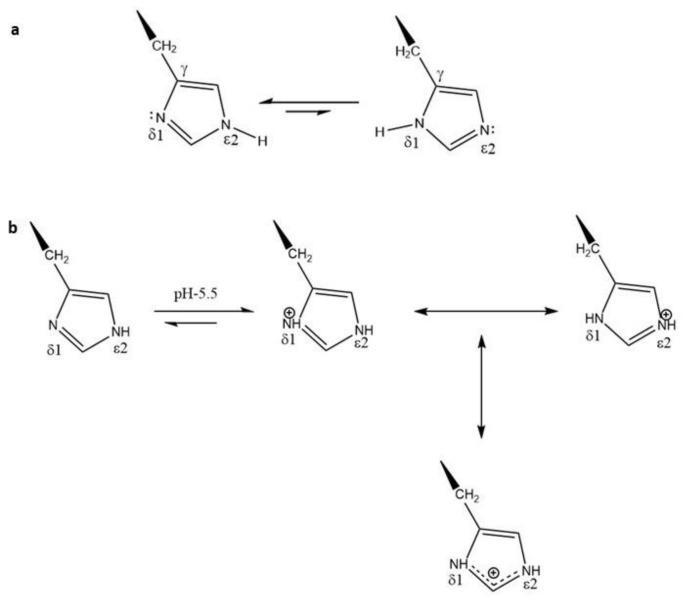
Tautomers of histidine within a peptide. (**a**) Non-equivalent tautomers of an unprotonated (neutral) histidine. The ε2N-H tautomer of the unprotonated histidine is the predominant tautomer compared to the δ1N-H tautomer. (**b**) Protonation of histidine with a pK_a_ of 6.0. At pH 5.5, histidine is primarily protonated (vs. the unprotonated form), and the positive charge is shared by the ring hydrogens of the two nitrogen atoms and the one carbon. At a higher pH (i.e., pH 7.4), histidine exists primarily in its unprotonated form.

With three carbons and two nitrogen atoms having an sp^2^ configuration, the imidazole ring of histidine is flat and aromatic. As the pH of the solution is raised, histidine becomes progressively less protonated. Moreover, the unprotonated (neutral) histidine exists as non-equivalent tautomers with a predominance of the ε2N-H (vs. δ1N-H) tautomer (Figure 1a) [5]. Whereas one nitrogen atom of the imidazole ring can act as a hydrogen acceptor, the other nitrogen atom acts as a hydrogen donor. Consequently, both nitrogen atoms can interact with other molecules through hydrogen bonding. The stabilizing properties of histidines within polyplexes are likely based on the aromatic and hydrogen bonds that the imidazole ring can form. The stability of the polyplex is greatly enhanced by the presence of histidines in the polymeric carrier, and this stability is likely important in the extracellular transit of the histidine-containing carrier. Compared to polylysine carriers of plasmids, histidine-containing carriers are significantly more stable to the effects of serum [6,7].

**Figure 2:**
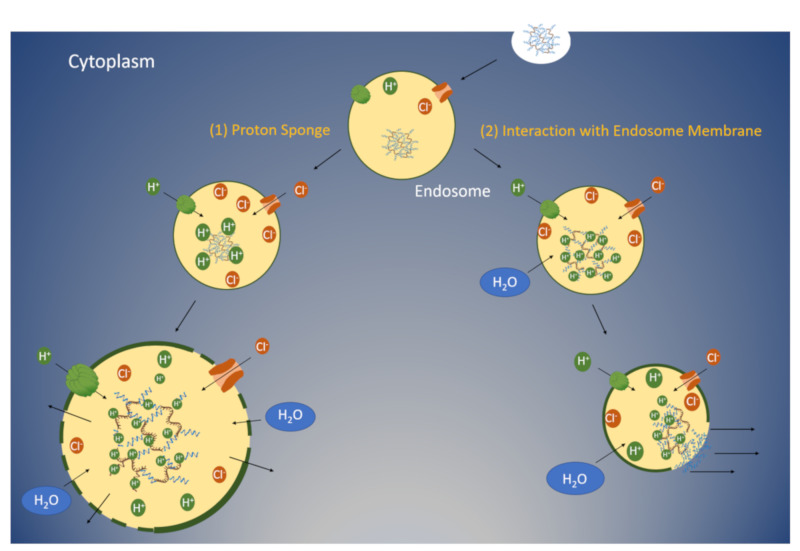
Two distinct mechanisms of endosomal lysis proposed for pH-sensitive carriers. The proton-sponge hypothesis is based on the buffering of acidic endosomes by pH-sensitive carriers, and active and then passive transport of hydrogen (H^+^) and chloride (Cl^−^) ions, respectively. This results in osmotic swelling and lysis of the endosomes. Alternatively, the release of the pH-sensitive carrier from the nucleic acids results in the carrier interacting with the endosomal membrane, enabling endosomal lysis. The range of pH in which these mechanisms occur varies widely (pH 5.0 to 6.5) [9].

The nitrogen atom on the imidazole ring with two unpaired electrons can accept hydrogens and buffer acidic endosomal environments between pH 5 and 7 (Figure 1b). The buffering of acidic endosomes by histidine-containing carriers is an important factor for the enhancement of nucleic acid delivery. It has been suggested that histidine-containing carriers, by neutralizing the pH, result in the active transport of protons with an influx of chloride ions to preserve neutrality in endosomes (Figure 2). This influx leads to passive transport of water into endosomes with osmotic swelling and lysis [8], enabling the escape of nucleic acids into the cytosol. An alternative mechanism for endosomal lysis is the interaction of the positively charged carriers with the endosomal membrane [9]. Upon acidification of the endosomes, the histidine-containing carriers become protonated. This protonation enables the charge-charge repulsion of the polymers, resulting in the break-up of the polyplex [10].

Although the histidine amino acid has a defined pK_a_ of 6.0, titratable histidines within peptides and proteins can vary widely [11,12,13,14]. Nevertheless, most histidines in cationic carriers of nucleic acids likely have a pK_a_ between 5.0 and 6.8 [11,12,14]. Even so, with this range of pK_a_, the differences in protonation of histidine are significant. Moreover, the pK_a_ of histidines between different polymers may not only vary, but the pK_a_ values of histidines within the same polymer may be markedly different [11,12]. This is due to the neighboring effect of adjacent amino acids to histidines. Ionic strength, polar, and non-polar interactions may influence the pK_a_ of histidines. Consequently, interactions of histidines in synthetic polymers with nucleic acids likely influence the pK_a_ of the imidazole ring. Notably, histidines that are buried within hydrophobic regions of micelles [15] or that interact with membranes may have pK_a_ which are quite different than the commonly stated pKa of 6.0 [11,13].

Several seminal discoveries led up to the development of histidine-containing carriers of nucleic acids (see reviews by Midoux et al. [16] and Lachelt et al. [17]). These developments include the addition of chloroquine to increase transfection [18] and the discovery of pH-buffering polymers which were effective transfection agents [19,20]. Perhaps the most relevant was the study by Midoux and colleagues that demonstrated histidine-containing peptides enhanced transfection [21]. The histidine-containing peptide, H5WYG (GLFHAIAHFIHGGWHGLIHGWYG), enhanced transfection of glycosylated polylysine DNA complexes by several orders of magnitude. Interestingly, the addition of serum (1% to 50%) to the media only modestly affected the transfection with H5WYG. Upon acidification of the media from pH 7 to 6.0, H5WYG underwent marked conformational changes, as evidenced by absorbance, fluorescence, and circular dichroism spectroscopy. The authors suggested that the enhanced transfection with the H5WYG peptide was due to its ability to permeabilize endosomal membranes at low pHs. Although H5WYG enhanced transfection, H5WYG was not associated with the polylysine complex. Nevertheless, as we will later discuss, the H5WYG peptide has been conjugated to virus-like capsids to enhance endosomal escape and siRNA silencing [22]. Importantly, the report by Midoux et al., stimulated several groups to develop histidine-lysine carriers of nucleic acids.

## 2. Histidine-Lysine Carriers of Nucleic Carriers

With the single repeating subunit-containing both DNA-condensing and pH buffering properties, polyethylenimine and polyamidoamine are efficient nucleic acid delivery agents. Except for altering the degree of branching, the binding and buffering properties of the two polymers cannot be readily varied. Consequently, there may be an inherent inflexibility of these polymers, which have a single repeating subunit with two or more functional features. In contrast, the ratios of histidines/imidazoles and lysines can be readily modified, affecting the ability of these histidine-lysine carriers to transport different forms of nucleic acids. Several studies have demonstrated the efficacy of nucleic acid carriers consisting primarily of histidines and lysines (Table 1). These histidine-lysine carriers or their polyplex cores may be further modified by PEGylation, cross-linkers including oxidized cysteines, or targeting ligands to enhance transfection.

**Figure 3:**
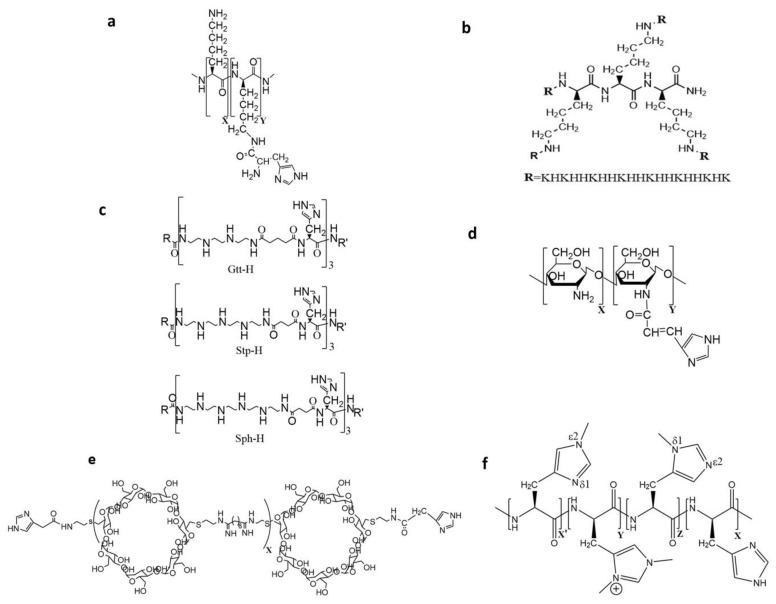
Chemical structures of selective histidine-rich polymers. (**a**) Histidylated polylysine, (**b**) four-branched histidine-lysine peptide with a 3-lysine core, (**c**) protonated and buffering subunits (Gtt-H, Stp-H, and Sph-H) incorporated into linear and branched polymers, (**d**) urocanic-modified chitosan, (**e**) cyclodextrin-containing polycation modified by two imidazole groups, (**f**) the imidazole ring of polyhistidine modified by methylation. The number of unmodified (X) and modified (Y, Z, X’) monomeric units in the polymer (**a**,**d**–**f**) are indicated.

In 1999, Midoux and Mosigny incorporated the histidine moiety into a polylysine transfection carrier (Figure 3a) to enhance gene expression in cell culture [6]. The average degree of polymerization (DP) for the histidylated polylysine was 190. They determined that the carrier was optimal when about 38% of the epsilon amino groups of polylysine were modified with histidyl residues. Compared to the polylysine carrier, the histidylated polylysine carrier increased plasmid (pDNA) transfection by more than a thousand-fold. Interestingly, the transfection of these histidylated polylysine polyplexes was not significantly affected by serum in the tissue culture medium. Moreover, the addition of bafiloymycin A1 markedly reduced transfection, indicating that the protonation of the histidines was essential for enhanced transfection [6].

Langer and colleagues provided further validation that conjugating greater numbers of imidazole groups to the polylysine template enhanced transfection [23]. The enhancement was non-linear in that the polylysine, in which 86.5% of its lysines were modified with imidazoles, had a 4-fold greater transfection than the one in which 73.5% of its lysines were altered. The polymer with the highest imidazole content had similar transfection efficiency as PEI but had significantly less cytotoxicity [23].

Although the transfection of plasmids was effectively delivered by the histidylated polylysine carrier with a DP of 190, this polymer was not an effective carrier of antisense oligonucleotides (ODN) [6]. After investigating several histidylated polylysines with lower DPs, Pichon et al. determined that a polylysine backbone with a DP of 19 was optimal. This histidylated oligolysine peptide in complex with the ODN markedly reduced the constitutively expressed ICAM-1 gene by 80% compared to the gene of untreated cells [24].

Similarly, the group of Seymour determined that smaller molecular weight reducible polycations (36–80 K, -CH_6_K_3_H_6_C-) were significantly more effective as a carrier of siRNA compared to the larger molecular weight polycation (162 K) [25,26]. By conjugating a liver-specific malaria circumsporozoite peptide to the lower molecular weight carriers of siRNA, further silencing of the hepatitis virus surface antigen was accomplished in liver-infected cells in vitro [25]. Reducible histidine-containing carriers were also effective carriers of plasmids [25,26,27]. In contrast to siRNA delivery, higher molecular weight reducible polycations were optimal carriers of plasmids [26]. Based on these and subsequent reports, we think that judicious use and placement of cross-linking cysteines stabilizes polyplexes and improves transfection with most polymeric carriers.

Furthermore, the transitional metal, zinc, has been noted to enhance the transfection of histidine-lysine polyplexes in cells [28,39,40]. At concentrations of 250 mM in the media, ZnCl_2_ improved GFP positive cells from 1% to more than 40%. This finding was selective to Zn^2+^ because no effect was obtained with other divalent cations. The mechanism was explored and fusion, and not permeabilization, was found to enhance the transfection of the polyplexes [28]. The addition of ZnCl_2_ directly to lipopolyplexes at lower amounts also enhanced transfection (1 μg per transfection, 1 μM in cell culture media) [39]. In this case, the chelation of zinc with the imidazole groups may stabilize the histidine-containing polyplexes or lipopolyplexes [39,40]. These coordination bonds are pH-sensitive so that zinc would be released inside acidic endosomes [41].

The earliest study to report the effectiveness of systemic delivery with HK peptide carriers of plasmids was done by Aoki et al. [29]. The peptide carrier, CRGDCF(K[H-]KKK)_6_ (cRGD-hK), was comprised of 36 amino acids and had three essential components: (1) a cyclic RGD which targets α_v_ integrins expressed on endothelial cells of tumors and on many tumor cells, (2) lysines which condense plasmid DNA, and (3) histidines, which buffer endosomes and aid in the escape of the plasmids into the cytosol. Twenty-five percent of the lysines were modified with histidines in the carrier. In the tumor-bearing mouse model, intravenous injection of the cRGD-HK/plasmid polyplexes was significantly higher in the tumor than in the lung, kidney, and spleen. Still, transfection in the tumor by these polyplexes was low, suggesting that further enhancement was required to obtain a reduction in tumor growth with tumor-inhibitory plasmids [29].

Although the polyhistidine-PEG conjugates can form polyplexes [10], the addition of cationic agents such as lysines increased the solubility of the polymer and polyplexes (particularly between the pH of 6 and 7.4), enhanced stability of the polyplexes, and increased DNA condensation and ultimately transfection. The importance of cationic lysines within the polymer was demonstrated by the group of Niidome in which histidines were conjugated to the terminal ends of the lysine dendrimer (KGH6). The lysines in the dendrimer were not charged, and the histidines on the surface of the dendrimer carried a greater positive charge at lower pHs. When formed at neutral pHs, the histidine-polyplexes had decreased compactness and low transfection efficiency compared to arginine-polyplexes. In contrast, when the histidine-polyplexes were formed at pH 5.0, they showed significantly higher transfection compared to when they were formed at neutral pH [30].

There were other considerations besides the total cationic charge on the polyplex to maintain solubility and transfection. The sequence patterns and the number of lysines and histidines can also affect the level of transfection or silencing activity [9,31]. For instance, a branched polymer with a repeating sequence of -KHHK- in complex with a plasmid has enhanced transfection compared to a repeating pattern of -KHK-[9]. Furthermore, removing a single lysine from N-terminal branches of an 8-branched carrier significantly attenuated its silencing activity [32]. These results suggested that there was a balance in the binding and release between the nucleic acid and the polymer in determining the efficacy of cytosolic import. These biophysical characteristics likely vary with the structure of the polymer. Although these studies indicated intermixing histidines and lysines in certain patterns of the branches enhanced transfection, other investigators have found that blocked histidine and lysine polymers were more effective as discussed below [38].

The group of Hanes determined that a triblock PEG-polyhistidine-polylysine (PEG-CH_12_K_18_) carrier augmented transfection in vitro and in vivo compared to the PEG-polylysine carrier (PEG-CK_30_). Despite the PEG-CH_12_K_18_ and PEG-CK_30_ nanoparticles having a similar size, worm-like structure, and zeta potential, the uptake of the two compacted DNA nanoparticles occurred by different pathways. Whereas uptake of the PEG-CK_30_ nanoparticle was dependent on nucleolin-dependent endocytosis (NDE), the uptake of PEG-CH_12_K_18_ occurred primarily by clathrin-mediated endocytosis (CME). Since CME occurred in almost all cells of the lungs and NDE occurred in one-third of the cells, the investigators reasoned that PEG-CH_12_K_18_ nanoparticles would be the preferred carrier. Delivered by the oropharyngeal route in mice, PEG-CH_12_K_18_ nanoparticles enhanced gene transfer to lung airways by about 3-fold compared to the PEG-CK_30_ nanoparticles. As demonstrated by cytokine levels, both nanoparticles showed minimal toxicity in vivo [33]. In an orthotopic glioblastoma mouse model, this group also demonstrated that PEG-CH_12_K_18_ was an effective carrier of an RNAi expressing plasmid (sh-Luciferase). Compared to untreated and plasmid treated groups, PEG-CH_12_K_18_ nanoparticles reduced luciferase expression in the glioblastoma tumors in vivo by about 60% ten days after their direct injection [34].

More recently, using a transfection assay with MDA-MB-435 malignant cells, our group demonstrated that branched H2K4b (Figure 3b) in complex with a luciferase-expressing plasmid resulted in high transfection in vitro, particularly at increased peptide: DNA ratios, where the nanoplex is positively charged. These and other malignant cells (4T1, MDA-MB-231) transfected with the linear H2K nanoplex had negligible luciferase expression at all peptide: DNA ratios [35]. In marked contrast to in vitro results, mice treated with linear H2K polyplexes, especially at low peptide: DNA ratios (~1:2 ratio), showed higher luciferase expression in xenografts and enhanced tumor specificity, compared to the H2K4b group [35]. The zeta potentials of linear or branched HK polyplexes at low peptide: DNA ratios (in water) were negative, ranging from −20 to −30mV. The relative expression of tumor: lung for H2K and H2K4b nanoplexes was ~2:1 and 1:14, respectively. The specificity toward tumors of these non-ligand, non-PEGylated H2K polyplexes may be due to these polyplexes intrinsically incorporating a tumor-targeting component. Because the neuropilin-1 receptor recognizes the -R/KXXR/K- sequence where X represents any amino acid [42], the H2K peptide with a repeating sequence of -KHHK- sequence is likely recognized by this receptor. Virtually all tumor endothelium and one-third of human tumors express NRP-1 receptor. Thus, the primary targeting mechanism of H2K nanoplexes was likely neuropilin-1 (NRP1)-mediated transport through the tumor endothelium. This conclusion was further supported by the marked reduction in gene expression of tumors from the H2K polyplex in mice pre-treated with the NRP-1 blocking antibody [36]. More evidence for NRP-1 mediated transport would have been obtained if gene expression of an HK peptide with a sequence other than -KHHK- was not blocked by the antibody. Further modifications of the H2K polyplex including PEGylaton and cRGD enhanced the specificity of the polyplex to the tumor xenograft [36]. Based on preliminary results, we also anticipate that the addition of cysteines to these H2K peptides may augment transfection.

### E. coli Genetically Engineered Carriers

**Figure 4:**
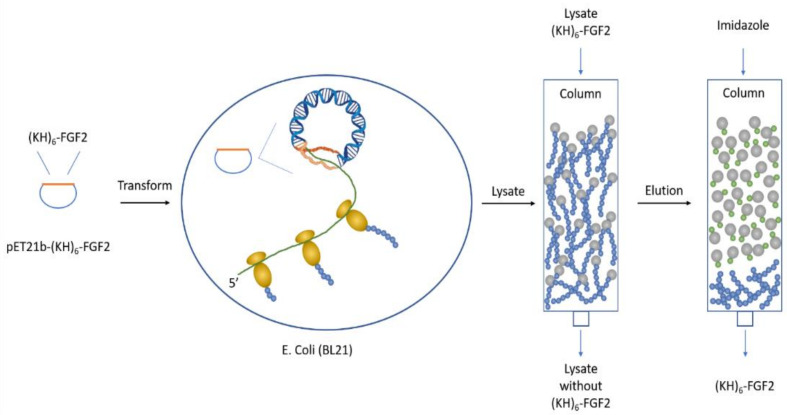
Schematic of purified histidine-lysine carriers which were biosynthesized by *E. coli* [37,38]. A plasmid encoding multiple repeats of HK peptides fused with FGF2 ((KH)6-FGF2) was used to transform *E. coli*. After the transformed bacteria were lysed, the soluble fraction containing the HK carrier was added to a Ni-NTA column, the column was washed to remove impurities, and then the peptide (KH)_6_-FGF2 was eluted from the column with an imidazole-containing buffer.

Unlike prior investigators who used solid-phase and solution-based syntheses of histidine-rich peptides, Hatefi et al. made HK carriers which were biosynthesized by *E. coli* (Figure 4) [37]. Multiple repeats of HK peptides ((KHKHKHKHKK)_6_) fused with FGF2 were purified by Ni-NTA column from the soluble fraction of the bacteria. This targeted HK carrier (diffuse or dKH-FGF2) could transfer plasmids effectively to various cell lines, including NIH 3T3, T-47D, and COS-1 in serum-free medium but not in medium with serum. In the presence of an excess of FGF-2, the transfection of FGF-2- containing HK polyplexes was markedly decreased, demonstrating that the mechanism was receptor-mediated endocytosis [37]. There was no evidence that the FGF-2 ligand had a role in enhancing the stability of the polyplex, and thus, it is likely that smaller peptide- targeting ligands could be substituted for the FGF-2 ligand.

Building on these results, a biosynthetic peptide with a cluster pattern of histidine and lysine sequences ((KKKHHHHKK)_6_-FGF2, cKH-FGF2) was compared for its ability to transfect cells with the initial biosynthetic HK carrier described above (dKH-FGF2) [38]. The clustered HK peptide was 5-fold more effective in transfecting cells compared to dKH-FGF2. Unlike the original peptide discussed above, the cluster cKH-FGF2 carrier maintained a high transfection in the presence of serum [36]. Of particular interest, even at a low N/P ratio (1:1), a stable polyplex with a large molecular weight targeting ligand (i.e., FGF-2) was made using the cluster HK peptide. The advantage of these biosynthetic methods is that high molecular weight linear HK peptides with specific patterns can be made that could not be readily synthesized with a peptide synthesizer.

## 3. Beyond H-K Peptides and Polymers

In addition to lysines, other amino acids and modifications have been incorporated into histidine-rich peptides and polymers to improve these carriers of nucleic acids (Table 2). Amino acids such as leucine, alanine, ornithine, and arginine, as well as non-amino modifications including diethylamines or amino ethyl modified histidines, have been used.

### 3.1. Sequence-Specific Histidine Carriers: Supplements and Alternatives to Lysines

Derived from mechanistic studies of antimicrobial peptides, LAH4 and its derivatives have proved effective carriers of plasmids and siRNA [43,44]. LAH4 is a cationic amphipathic peptide containing lysines, leucines, alanines, and histidines (LAH4: KKALLALALHHLAHLALHLALAL- KKA). After sequence modifications of LAH4, the authors concluded that the number and placement of histidines were essential for efficient cytosolic delivery of nucleic acids. In four cell lines, the amphipathic cationic LAH4 peptide had a similar transfection profile as PEI [43]. Interestingly, the LAH4 peptide has proven effective as a carrier of peptides and proteins in addition to plasmids and siRNA [50]. When injected subcutaneously, the LAH4 carrier of a peptide fragment of tyrosine-related protein-2 and a CpG oligonucleotide showed an enhanced prophylactic antitumor specific CD8+ T cell response against B16 murine tumors [51]. The amphipathic LAH4 nanoparticles can vary from 100 nm to a few microns in size [50].

When the close analog, ornithine, was substituted for lysine, the ornithine-histidine was demonstrated to be more effective than the lysine-histidine carrier [45]. Charmathy and colleagues compared three sequence-defined peptides made up of 16 amino acids for their ability to carry plasmid DNA into dendritic cells. In contrast to polylysine (K_16_) and the polylysine-histidine (K_10_H_6_) polymeric carriers, the blocked polyornithine-histidine (O_10_H_6_) copolymer had significantly higher transfection efficiency with reduced toxicity to DC cells [45]. Additional studies by this group demonstrated coating a microsphere with O_10_H_6_ carrier greatly enhanced the delivery of ODNs into DCs [46]. Furthermore, when DC cells were transfected with O_10_H_6_-plasmid polyplexes encoding interleukin 10, the host response to Matrigel impregnated with the modified DC cells was greatly suppressed in a host vs. graft mouse model [52].

To enhance endosomal lysis and cytosolic import of nucleic acids, different patterns of ten histidines were incorporated into the TAT peptide (RK_2_R_2_QR_4_) [47]. The TAT peptide, an arginine-rich peptide, is known to enhance the cellular uptake via macropinocytosis of a large variety of molecules including polyplexes. Lo and Wang determined that the optimal transfection carrier of DNA was the H_5_-TAT-H_5_ peptide, a carrier in which five histidines were on both the N- and C-terminal ends of TAT. There was a significant improvement in gene transfection efficiency in vitro with this modified TAT peptide (H_5_-TAT-H_5_) compared to the unmodified TAT peptide. Moreover, by incorporating two cysteine residues into the H_5_-TAT-H_5_ design, the peptide (C-H_5_-TAT-H_5_-C) was more effective, probably because the disulfide bonds formed multimers, which enhanced stability of peptide/DNA complexes [47]. The C-H_5_-TAT-H_5_-C carriers resulted in lower gene expression than PEI-25kDA after intrastriatum injection, but these carriers resulted in comparable expression with intrathecal injections.

Taking a different tack to develop sequence-specific carriers, Lachelt et al., examined several linear and branched cationic oligoethanamino amide as carriers of plasmids [48]. Instead of lysines that bind to nucleic acids [6,9,32], the cationic segments of these oligoethanamino amide polymers were made up of diaminoethanes, the same repeating motif that is in PEI. One potential advantage of diaminoethanes with a higher charge density than lysines is enhanced stability of the polyplexes. Together with histidines, the different oligoethanamino building blocks varying in size and protonable nitrogens (succinoyl pentaethylene hexamine, Sph; succinoyl tetraethylene pentaamine, Stp; glutaroyl-triethylene tetramine, Gtt) provided a cationic charge necessary for binding to DNA and a buffering component over a wide range of endolysosomal pH to enhance transfection in vitro and in vivo (Figure 3c). Whereas histidine markedly enhanced transfection of the four-branched polymers comprised of the larger building blocks, Sph and Stp, the addition of histidines had little effect on the transfection of the low molecular weight Gtt building block. The authors reasoned that lack of transfection enhancement by histidines was based on the inability of Gtt polymers to bind DNA. To increase the molecular weight of the four-branched Gtt polymer and its ability to bind DNA, a cysteine was added which resulted in an increase in transfection. Notably, the 4-branched histidine-containing Sph polymer was 20- to 80-fold more effective than the branched sequences of H2K4b [9]. Furthermore, endosomal lysis and transfection of targeted PEGylated oligoethanamino polyplexes were markedly enhanced compared to non-targeted polyethylene glycol polyplexes.

Similarly, the group of Wagner examined several c-Met targeted cationic oligoethanamino amide polyplexes [49]. In contrast to their initial study, targeted PEGylated and non-PEGylated polymers were combined to form a stable plasmid polyplex which was resistant to degradation by high serum levels (90%) and heparin. The optimal coformulation formed either nanorods of about 100–150 nm or toroidal structures of about 50 nm. Moreover, both the targeted PEGylated and non-PEGylated polymers contained cysteines that increased stabilization of the polyplex and enabled the release of the plasmid in the high reducing environment of the cytosol. The combination of targeted PEGylated and non-targeted polymer, in which histidines and cysteine were incorporated, enhanced expression of plasmids in vitro and in vivo. Enhanced transfection of cells with luciferase-expressing plasmids was also found with sequence-defined histidine-rich oligoaminoamides and a transferrin-PEI conjugate [53]. Nevertheless, not all studies determined that histidine-containing oligoethanimo amide polymers increased nucleic acid delivery to their target. For example, the addition of histidines to a lipooligopeptide carrying the siEG5-KLK apoptotic conjugate did not enhance the anti-tumor activity of the nanoparticle [54].

### 3.2. Histidine/Imidazoles Added to Parent Polymers

There are several examples of histidine/imidazoles or histidine-rich peptides added to parent carriers to enhance the delivery of nucleic acids (Table 3). Investigators have added histidine/imidazole-containing peptides to the parent non-biodegradable and biodegradable carriers for several reasons. For non-biodegradable carriers, an unheralded role of histidines is its ability to reduce the toxicity of polymers and polyplexes [55].

#### 3.2.1. Non-Biodegradable Polymers

Unlike polysaccharide carriers discussed below, the commonly used polyethylenimine (PEI) polymers are toxic to cells at low concentrations. Considerable effort has been undertaken to minimize the toxicity of these polymers. Since toxicity is related in part to the non-biodegradability of these polymers, reducing their molecular weight and degree of branching can decrease their toxicity. Alternatively, modification of these dendrimers with histidines has been shown to mitigate their toxicity [55,56,57,67,68]. By conjugating imidazoles to branched PEI, reduced toxicity of the modified PEI was observed in vivo and was mediated by a decrease of cytokines, chemokines, and reduced liver injury [68].

Using the Michael reaction, Bertrand and colleagues prepared a number of histidine-linear polyethylenimine conjugates (H-lPEI). The conjugates with the greatest buffering capacity between pH 5 and 7.4 showed the highest transfection efficiency, with up to 95% of the cells transfected. Notably, polyplexes of these conjugates showed very low cytotoxicity compared to the unmodified linear PEI (IPEI) polyplexes [55]. More recently, the group has combined lPEI and H-lPEI to improve transfection in fibroblasts and muscle cells. Specifically, the combination of lPEI/H-lPEI containing 57% and 67% IPEI had transfection similar to lPEI, yet this combination showed low cytotoxicity comparable to H-lPEI [56]. Although the authors speculated that the reduced toxicity of H-lPEI was due to the carboxyl groups (on the histidines), it would be interesting if imidazoles (without the carboxyl groups)-lPEI conjugates were compared to H-lPEI [55].

Oligopeptides incorporating histidines and arginines (or guanidium groups) also have increased the efficacy of other carriers. When histidines and arginines were coupled to generation 4 of PAMAM (PAMAM G4), the transfection efficiency and buffering capacity of the modified dendrimer was significantly enhanced. As the number of histidines in the peptide (3, 2, or 1) conjugated to the dendrimer increased, the transfection activity of the dendrimer was enhanced (i.e., PAMAM His3-Arg > PAMAM His2 Arg > PAMAM His-Arg > PAMAM-Arg). Besides having higher transfection, the PAMAM-His3-R had less toxicity than PEI25kD [57]. Nevertheless, since PAMAM dendrimers are not degradable, we have concerns about the long-term safety of these dendrimers.

#### 3.2.2. Biodegradable Polymers

##### Polysaccharides

Although cationic chitosan is biodegradable and has low toxicity, the transfection with chitosan is low. To enhance its transfection, chitosan was conjugated with urocanic acid bearing an imidazole ring (Figure 3d). With gel retardation assays, the chitosan-urocanic conjugate bound DNA more tightly than the chitosan alone carrier. Furthermore, the transfection efficiency of the urocanic-modified chitosan carrier was increased in cells by 10-fold compared to the unmodified chitosan carrier [58]. The authors then carried out two in vivo studies demonstrating that an aerosolized chitosan conjugate in complex with tumor suppressor genes markedly reduced the development of lung tumors [59,69]. For instance, after eight treatments with the chitosan conjugate in complex with a plasmid expressing PTEN, the number of lung tumors in a K-ras mouse model was reduced by about 55% compared to the untreated controls. Repeated injections of the chitosan-urocanic conjugate carrier were well-tolerated with low toxicity.

In addition, modification of chitosan with an arginine-histidine blocked copolymer (R_6_-H_6_) was determined to enhance the systemic delivery and anti-tumor activity of a siRNA targeting survivin [60]. Whereas R_6_-H_6_ CS carrying survivin siRNA inhibited the 4T1 mouse breast tumor cells by 46%, the unmodified CS reduced cell numbers by 28% (*p* < 0.05). Perhaps most impressively, despite the large size of the tumors upon initiation of the therapy, the R_6_-H_6_ CS carriers of siRNA inhibited tumor size by about 50% after the sixth treatment compared to the untreated group. The decreased size of the primary tumor correlated with reduced metastases to the lungs and liver as well as prolonged survival compared to the naked siRNA treatment group. A chitosan carrier in which only polyarginine was conjugated to its surface would have been a helpful control (R_6_-CS) in judging the efficacy of the histidine component of R_6_-H_6_ CS in vitro and in vivo [60]. In another study, when multiple sites of chitosan were modified with only histidines, transfections in vitro and in vivo were only modestly increased [61]. The increased charge and buffering capacity with the addition of arginine and histidine copolymers were likely necessary to increase the transfection efficacy of chitosan. Thus, a balance between positive charges and pH-buffering components was required for efficient transfection.

To increase the buffering capacity, two buffering groups were incorporated within the cyclodextrin carriers [62] (Figure 3e). When compared to the cyclodextrin-containing polycation (CDP), the imidazole-containing CDP (imCDP) displayed a number of mechanistic differences in its delivery of nucleic acids. Whereas imCDP bound nucleic acids (including oligonucleotides, siRNA, and plasmids) more tightly than CDP as demonstrated with cell-free assays, the imCDP released nucleic acids more readily intracellularly. These findings were consistent with other studies showing that the protonation of histidines within the acidic endosomes would aid in the release of nucleic acids. Besides the ability to lyse endosomes, the enhanced extracellular stability and release of the nucleic acids had a role in imCDP having a greater transfection efficiency compared to CDP [62].

The cationic curdlan, another polysaccharide, was modified to varying degrees with imidazoles [63]. The modified polymers with the highest percentage of imidazole (~25%) had the highest endosomal escape and transfection efficiency. The optimal polymer transfected nearly 56% of the 293T cells, whereas the unmodified polymer transfected only 24% of the cells. The imidazole modified curdlan formed nanoparticles with nucleic acids in the range of 80–105 nm. These modified polymers were quite effective as carriers of siRNA as demonstrated by the nearly 60% silencing of their target. In contrast to transfection, the silencing studies did not correlate with the degree of imidazole modification of these polymers [63].

##### Alternative Biodegradable Carriers

The imidazole-containing phosphophazenes have similarities to the polylysines modified by histidines [6] or imidazoles [23] discussed previously. For instance, the cationic charges and imidazole groups were randomly dispersed on the biodegradable polyphosphazenes. Incorporating imidazoles and the cationic 2-dimethylaminoethylamino (DMAEA) as side groups onto the biodegradable polyphosphazene (PIDP) enhanced transfection efficiency and reduced toxicity in several cell lines compared to the non-imidazole polyphosphazene (PDAP) control. Whereas the half-life of PIDP degradation at pH 7.4 was 22 days, the half-life was three days at pH 5.0. The PIDP carrier, which had a histidine content of 17%, formed plasmid nanoparticles of about 100 nm in size at a 10:1 ratio [64]. Carriers with higher imidazole content (i.e., 41%, 69%) formed large polyplexes greater than 300 nm and were not used for transfection experiments.

The addition of imidazoles/histidines to polymers did not always enhance transfection. For instance, end-capping of poly(β-amino esters) polymer, C32, with histidines had a lower transfection efficiency than end-capping with the more hydrophilic primary and tertiary amines [65]. The location and low numbers of imidazoles were the likely reasons for the reduced transfection by these imidazole-modified polymers.

Since most aptamer-siRNA chimeras are degraded in the lysosomal pathway, efforts have been made to increase endosomal lysis, enabling the escape of the siRNA intracellularly. One such strategy was to fuse a PSMA aptamer with a double-stranded RNA binding domain and polyhistidine. The double-stranded RNA domain binds one or two siRNA, and the polyhistidine enables endosomal lysis [66]. The addition of an 18-mer of polyhistidine was optimal in augmenting endosomal escape and increased gene silencing by about 5-fold in LNCaP cells. Interestingly, the addition of histidine-tags of 24 or 30 amino acids inhibited siRNA binding to the RNA-binding domain. The authors suggested that these chimeras remained discrete, enabling the chimeras to permeate deeply into tumor tissues.

### 3.3. Modification of Polyhistidines

With the pH above 6.0, the solubility of polyhistidine is greatly reduced [70] and the stability of such polyplexes remains a challenge. Thus far, we have discussed several strategies that have incorporated lysines or other cationic agents with histidines/imidazoles in carriers to bind DNA and increase the solubility of the carrier and the polyplex. Alternatively, there have been approaches to enhance the solubility and stability of polyhistidine polyplexes, particularly when these are formed at neutral pH (Table 4).

In early 2000, the group of Langer was the first to report about modifying polyhistidine for transfection studies [71]. They conjugated gluconic acid with polyhistidine which resulted in a partially glycosylated polyhistidine polymer, soluble at pH 7.4. Although the glycosylated polyhistidine could complex DNA based on the gel retardation and dye exclusion assay, polyhistidine was not an effective transfection carrier, perhaps due to the large size of the polyplex. Nonetheless, a ternary complex, which was comprised of glycosylated polyhistidine, transferrin-targeted polylysine, and plasmid DNA, showed significant enhancement in transfecting cells compared to the binary polylysine-plasmid DNA polyplex.

To increase solubility and avoid interaction of polyplexes with blood components, the group of Langer later attached polyethylene glycol (PEG) to polyhistidine. Both comb-shaped and linear A-B block copolymers were synthesized with PEG and polyhistidine [10]. Interestingly, despite the lack of lysines, some of the formulations of these polyplexes, even at a neutral pH in which a low percentage of histidines were protonated, were able to condense plasmid DNA and were relatively stable (i.e., size) for up to a week. Nevertheless, most of these polyplexes had greater stability under acidic conditions. Moreover, despite a negative zeta potential suggesting that the DNA was present on its surface, the polyplexes with the linear-AB block polymers were also able to resist DNAse I digestion. As expected, transfection of these PEGylated histidine polyplexes was low, and the authors proposed in future studies to conjugate ligands to these polyplexes to enhance transfection.

Instead of using a separate cationic agent or PEG, Asayama et al. modified polyhistidine by amidation or methylation to affect the stability of the nanoparticle or its ability to bind to DNA. Initially, they added aminoethyl groups to the imidazole ring which increased its hydrophilicity and its binding to DNA [72]. Compared to polylysine, the amidated polyhistidine carrier modestly enhanced plasmid transfection. With 15 mole percent of polyhistidine modified by aminoethyl groups, turbidity of the amidated polyhistidine occurred between pH 6 and 7. This suggests that modification of polyhistidine with higher percentages of aminoethyl groups may have mitigated the solubility issues and enhanced transfection.

In a later study, the team of Asayama modified the histidine ring with methyl groups which led to an increase in the transport of nucleic acids into cells (Figure 3f). By modifying polyhistidine with methyl groups, they investigated whether modified polyhistidine carriers with different percentages of methylhistidines (δ1-N or ε2-N), dimethylhistidines, and unmodified histidines enhanced transfection. Whereas the dimethylhistidines increased the positive charge at neutral pH, the methyl histidines and unprotonated histidines affected the hydrophobicity and buffering capacity of the polyhistidine carrier. The modified polymers were classified into 3 categories based on their dimethylimidazolium content: (1) PLH-Me (25 mol%), (2) PLH-Me (68 mol%) and (3) PLH-Me (87 mol%) [73,74]. The PLH-Me (25%) was the most effective carrier for both siRNA and plasmids, whereas the PLH-Me (87%) was the least. Gene silencing or expression depends on the optimal content balance among hydrophobic, buffering, and cationic groups. Moreover, although PLH-Me (25%) was less efficient as a carrier than PEI, the PLH-Me polyplex was significantly less toxic to cells.

By modifying polyhistidine with carboxymethyl groups, the transfection of cells with a ternary polyplex was significantly enhanced [75,76]. After preparing a positively charged PEI-plasmid polyplex, the negatively charged carboxymethyl-polyhistidine was added to coat the polyplex. In addition to altering the surface charge, the carboxymethyl groups enhanced the solubility of polyhistidine at physiologic pH. The ternary polyplex increased transfection more than 300-fold compared to the binary PEI polyplex. Based on this research [75], Sha and colleagues prepared ternary polyplexes that significantly enhanced transfection to tumors in vivo [76]. The ternary polyplexes were comprised of plasmids, carboxymethyl polyhistidine, and poly (β-amino ester) polymers. The in vivo gene transfection study demonstrated that the ternary polyplexes enhanced luciferase expression by 4-fold in tumors compared to binary poly (β-amino ester) polymer-plasmid polyplexes [76].

## 4. Lipopeptides

Lipopeptide or lipid-peptide hybrid carriers form micelles alone and in complex with nucleic acids (Table 5). Since the earliest report of lipid-peptide hybrids, these hybrids have evolved into an important area for polymeric carriers [77], in part because of their ability to increase the stability and/or the half-life of the polyplexes in the bloodstream [78,79,80].

Porosk et al., prepared a series of histidine-containing lipopolymers to optimize siRNA delivery [81]. Previously, the group modified KALA containing lipopolymers with a pH-buffering chloroquine analog to enhance siRNA delivery [82]. Because of the potential toxicity in vivo of the chloroquine derivative, histidines were substituted for the chloroquine modification. Particularly striking was the finding that minor modifications in these histidine patterns resulted in significant differences in the stability and silencing activity of these carriers. The silencing effect, the stability, the membrane activity at several pHs, and the partition coefficient of the polyplexes enabled the selection of the optimal polymer. They determined that some histidine patterns that were N-terminal to the KALA group were effective carriers in vitro and in vivo. After a single intravenous injection, one histidine-containing analog, H6-20 (also named NickFect70), silenced factor VII in the liver of mice by about 60% compared to untreated controls [81]. Chen et al. demonstrated that a stearylated histidine-containing cationic peptide in complex with siGADPH was as effective as silencing its target in vitro as lipofectamine [83]. This study indicated that the addition of histidines enhanced the transfection efficacy of the lipopeptide.

Recently, Li and colleagues co-delivered in micelles a siRNA targeting PD-1 and methyl-tryptophan to reduce tumor size. Both the siRNA and methyl-tryptophan, a small molecule inhibitor of indoleamine 2,3-dioxygenase, targeted dual immune checkpoints, thereby enhancing the potential to overcome tumor immune resistance. The micelle was composed of a NRP-1 tumor-targeting peptide (AKRGARSTA), a histidine-rich domain, and cholesterol. As discussed previously, the NRP-1 receptor recognizes the -RGAR- motif. The micelle specifically accumulated in the 4T1 breast cancer tumor compared to other tissues. Moreover, the micelle, which incorporated the siRNA and inhibitor, markedly reduced tumor size. Compared to either checkpoint inhibitor, co-delivery of the siRNA and 1-methyl-dl-tryptophan increased survival of cytotoxic T lymphocytes, resulting in apoptosis of cancer cells. The probable role of the histidine-rich domain of the micelles enabling the siRNA to escape from the endosomes was demonstrated by confocal fluorescent microscopy [84].

**Table 5:** Lipopeptide and liposome-polymer carriers for nucleic acids.

Polymer	Nucleic Acid	In Vitro/ In Vivo	Comment	Reference
**LIPOPEPTIDES**				
H6-20 (NickFect 70, a stearylated peptide)	siRNA	Y/Y	Several analogs tested that varied in amino acid sequence and fatty acid length; a histidine-rich stearylated peptide effectively silenced the target gene both in vitro and in vivo.	[81]
Stearylated histidine-containing cationic peptide	siRNA	Y/N	Stearylated-HHHPKPKRKV ^1^ peptide in complex with siRNA was as effective as silencing its target in vitro as lipofectamine	[83]
Chol-HHHHHHHAKRGARSTA	siRNA	Y/Y	NRP-1 targeted peptide hones micelle toward tumor. Micelle incorporated siPD-L1 and 1-methyl-DL- tryptophan, which provided dual blockade of checkpoints for breast cancer.	[84]
**POLYMER-LIPOSOME**				
Linear HK/liposome	pDNA	Y/Y	Co-polymer increased 100-fold transfection compared to liposome alone in serum in vitro. Also increased 15-fold the activity of luciferase expression in tumors in vivo.	[85]
H2K4b/liposome	pDNA	Y/N	Branched histidine-lysine peptide and liposomes markedly increased luciferase expression compared to Linear HK-liposomes in malignant cells.	[86]
hK-Liposome	pDNA	Y/Y	The hK peptide, K[K(H)KKK]_5_-K(H)KKC, was conjugated to cationic liposomes. Improved chemosensitivity in vitro. Targeted hK liposomes increased delivery in vivo by 3-fold compared to targeted non-hK liposomes	[87]
PEGylated histidylated polylysine (PEG-HpK)/histidylated liposome (His-Lip)	mRNA	Y/Y	Carrier of MART1/MART1-LAMPI mRNA injected iv induced CD8^+^/CD4^+^ T cell response. Mice treated with optimal vaccine prophylactically had reduction in B16 tumor size	[88]
PEG-HpK/Mannosylated (Man)- imidazole- histamine-Lip (Im-Hist-Lip)	mRNA	Y/Y	Mannosylated carrier injected iv expressed EGFP 4 times in DCs of spleen than sugar-free carrier. This improved delivery correlated with better inhibition with MART1 vaccine.	[89]
PEG-HpK/Tri Man- Im-Hist-Lip	mRNA	Y/Y	Tri-mannosylated carrier LPR with E7 mRNA had potent antitumor activity with improved safety profile compared to lipoplex	[90]
H-lPEI/Im-Hist-Lip	siRNA	Y/N	Potent carrier of siRNA with low cytotoxicity	[91]

^1^ One amino acid code; chol, cholesterol; NRP-1, neuropilin-1; PD-L1, program cell death ligand 1; H2K4b, four-branched histidine-lysine polymer; PEG-HpK, PEGylated histidylated polylysine; Man, Mannosylated; Tri-Man, Tri-mannosylated; H-IPEI, linear polyethylenimine containing 16% histidines; Im-Hist-Lip, imidazole-histamine-Liposome.

## 5. Lipopolyplexes/Lipoplexes

Histidine-rich peptides combined with cationic liposomes (lipopolyplexes or lipoplexes) are closely related to histidine-rich peptide-lipid hybrids (Table 5). Lipopolyplexes are ternary complexes formed between nucleic acids, a cationic polymer, and liposomes. When protamine was first complexed with DNA before the addition of cationic liposomes, transfection with the lipopolyplex was markedly enhanced [92].

Our group adopted a similar strategy to form lipopolyplexes due to the low transfection of the linear HK peptide [85]. As a result, we determined that the combination of the linear HK/liposome/DNA lipopolyplex increased transfection as much as 10-fold in comparison with the liposome/DNA lipoplex. In the presence of serum, the linear HK lipopolyplex increased transfection efficiency up to 100-fold. Furthermore, in vivo expression of luciferase in a tumor increased 15-fold with the linear HK lipopolyplexes [85]. In a subsequent study, gene expression was much higher with branched HK than linear HK lipopolyplexes in malignant cells [86]. We discuss these results further in the mechanistic section of this review.

Another peptide discussed earlier, hK [29], inspired an improved liposome carrier of nucleic acids. Yu et al. conjugated a histidylated oligolysine (hK) with targeted cationic liposomes to improve plasmid delivery [87]. The hK-containing cationic liposomes improved the delivery of plasmid both in vitro and in vivo. Moreover, a p53-expressing plasmid with the targeted hK liposome greatly enhanced the sensitivity of the p53-deficient prostate tumor cells (DU145) to mitoxantrone in vitro. Promising results were also observed in vivo with systemic delivery of these hK targeted lipoplexes to prostate tumors. Without affecting gene expression in normal tissues, these lipoplexes delivered improved GFP plasmid delivery to tumors by 3-fold compared to targeted non-hK liposome carriers [87].

Other investigators have focused on histidylated lipopolyplexes for their vaccine and silencing potential [16,88,89,90,91]. After initially synthesizing lipids with various headgroups, the group of Midoux determined that the histidine-containing (His-Lip) and later imidazole/histamine- containing (Im-Hist-Lip) liposomes were most effective in gene transfer. The group then reported in several studies the effectiveness of imidazole-containing liposomes in combination with a histidylated polymer as a carrier of plasmids, mRNA, and siRNA [16,88,89,90,91].

PEGylated histidylated (PEG-HpK) lipopolyplexes (LPR) effectively delivered melanoma-associated antigen MART1 mRNA systemically for an anti-tumor prophylactic response in a mouse model. Because of the improved efficacy of an mRNA vaccine compared to a DNA vaccine, mRNA expressing the MART1 vaccine was selected. The anti-tumor effect was significantly greater with MART1 mRNA carrying a poly(A) tail length of 100 adenosines compared to a poly(A) tail length of 64 adenosines. Interestingly, the anti-melanoma response was further enhanced by incorporating both MART1 and MART1-LAMP1 mRNA (MART1/MART-LAMP1) into the polyplex. The MART1 mRNA and MART1-LAMP1 mRNAs likely targeted MHC class 1 and MHC class 2, respectively. By inducing CD4+ and CD8+ T lymphocytes, the MART1/ MART-LAMP1 mRNA lipopolyplex vaccine reduced tumor growth by about 75% [88].

To further enhance the anti-tumor RNA vaccine, mannosylated-targeted lipopolyplexes (Man-LPR) were prepared to increase the uptake of MART1 mRNA by dendritic cells (DCs) [89,93,94]. After the PEG-histidylated-polylysine (PEG-HpK) RNA complexes were made, they were added to the mannosylated and imidazole-histamine liposomes (Man-Im-Hist-Lip) to form the ternary lipopolyplex (Man-LPR). When the lipopolyplex with GFP mRNA were injected systemically in a mouse model, the expression of GFP was 4-fold higher in splenic dendritic cells than with the sugar-free mRNA lipopolyplex (LPR). Consistent with the result, the anti-tumor prophylactic response with MART1 mRNA was significantly greater with the Man-LPR. Specifically, the mean tumor volume at day 21 was about 200 mm^3^ in those treated with the Man-LPR vaccine, whereas the volume was about 500 mm^3^ in the sugar-free carrier mRNA vaccine [89].

Recently, decorating the liposome surface with a “tri-antenna of α-D-mannopyranoside” (Tri-man) improved the specificity toward dendritic cells compared to those decorated with mannose [95]. In another report, Tri-Man LPR vaccine targeting E7, injected intravenously, enhanced the antitumor response in a therapeutic mouse model and improved the safety inflammatory profile compared to the lipoplex [90]. With intradermal injections, the TPR-Man vaccine, directed against different antigens in three therapeutic tumor models, showed marked antitumor activity. This platform has significant promise as a delivery system for therapeutic cancer vaccines in humans [96].

The histidylated linear lPEI (H-lPEI) LPR carrier was demonstrated to be less toxic and a more effective carrier of siRNA than several common commercial carriers [91]. Whereas the H-IPEI polymer was not an effective carrier of siRNA, the H-lPEI-LPR reduced GFP expression in HeLa cells by about 70%, forty-eight hours after transfection. Moreover, at low siRNA concentrations (10 nM), the H-lPEI LPR silenced GFP about 2 1/2-fold more than DOTAP LPRs (65% vs. 25%). Based on this and prior studies, histidylated lipopolyplexes seem to be effective carriers for DNA, mRNA, and siRNA.

## 6. Pre-Formed Nanoparticles and Virus-Like Particles Modified with Imidazoles

In addition to liposomes, non-liposomal preformed vectors have been modified with imidazoles/histidines or histidine-rich peptides as carriers of nucleic acids. These preformed nanoparticles include mesoporous silica particles and viral-like particles (Table 6). Histidylated/imidazole altered lipids in which liposomes or micelles are the sole carriers of nucleic acids will not be discussed (see review by Midoux et al. [16] and Gujrati et al. [97]).

To augment the delivery of plasmids in vitro and in vivo, Brevet et al., first modified mesoporous silica nanoparticles (MSN) with aminopropyltriethoxysilane (MSN-NH2) and then with L-histidine (MSN-His) [98]. The authors noted that prior modifications of MSN with cationic polymers such as PEI had resulted in considerable toxicity at low concentrations. Compared to MSN-NH2 carriers, MSN-His showed better protection from DNA degradation, improved endosomal escape, and reduced cytotoxicity. Increased transfection efficiency was also observed in cells in vitro and in the Achilles tendon in vivo [98].

In another report, imidazoles were conjugated via a Schiff base linkage to mesoporous silica nanoparticles to enhance the delivery of survivin shRNA-expressing plasmids and doxorubicin to tumors. The addition of the pH buffering imidazoles to MSNs had two goals other than endosomal lysis. One, the presence of imidazoles should enhance the delivery of MSN to solid tumors such as hepatomas because of the pH gradient between the blood with a pH of 7.4 and acidic tumors with a pH of about 6.9. Because of the pH gradient, this selectivity for acidic solid tumors will likely be observed for most nanoparticles containing histidines or imidazoles on their surfaces. Second, the pH-sensitive Schiff-base likely aided in the release of survivin-shRNA and doxorubicin from the MSN in the acidic endosomes. The imidazole MSN nanoparticles had a size of about 327 nm. Despite the large size of the imidazole MSN nanoparticles co-delivering the survivin-shRNA and doxorubicin, the growth of the tumors was completely inhibited. Moreover, systemic delivery of these histidine-containing MSN reduced tumor size significantly more than non-histidine MSN (*p* < 0.01) [99].

Because of difficulty loading siRNAs into MSNs, Pan and colleagues used an ingenious zinc-imidazole framework (ZIF-8) of a few nanometers of thickness to cover the surface of the mesoporous silica nanoparticles [100]. The zinc-imidazole film blocked the pores of the MSN enabling efficient loading of siRNA, and its pH buffering ability facilitated the escape of the siRNA and doxorubicin from the MSN and endosomes. Furthermore, the ultrathin zinc-imidazole film decomposed within the acidic endosomes. Because Bcl-2 induces anti-apoptosis genes and proteins which results in resistance to doxorubicin, the efficacy of ZIF-8 MSN carrying doxorubicin and an siRNA targeting Bcl2 was examined. Together the siRNA and doxorubicin incorporated with ZIF-8 MSN were significantly more effective in inhibiting and in inducing apoptosis of doxorubicin-resistant cells compared to MSNs carrying doxorubicin alone [100].

To enhance siRNA delivery with viral-like particles (VLPs), the peptide H5WYG, which helped launch the field of histidine-modified carriers [21], was conjugated to the particles [22]. SiRNAs targeting several cyclins were efficiently incorporated within modified VLPs derived from bacteriophage MS2 to target hepatoma cells. In the presence of siRNA, the coat proteins of MS2 self-assembled into particles containing about 85 siRNA. The capsids were modified in precise locations via chemical conjugation, with hepatocellular targeting ligands (SP94), PEG, and an H5WYG endosomal escape protein. MS2 VLPs modified with SP94 exhibit a 10^4^-fold higher avidity for hepatocellular cancer cells than for hepatocytes. The SP94-targeted VLPs, which encapsidated a siRNA cocktail, effectively silenced expression of several cyclins and induced growth arrest and apoptosis of Hep3B cells. There were about seventy-five “H5WYG” peptides per particle to promote endosomal escape and enhance siRNA silencing. Without the histidine-rich fusogenic peptide, the MS2-containing siRNA was significantly less effective [22]. Consequently, similar strategies using histidine-rich peptides can be adopted to enhance uptake and endosomal escape for viruses or nanostructures such as nanotubes and nanospheres [50].

## 7. Mechanistic Insights from Histidine-Enriched Carriers and Polyplexes

Many studies have focused on the histidine-buffering properties and not on its many other properties. The study by the Davis group showed the multiple functions of imidazole-modified polymers, including increased extracellular stability and enhanced intracellular release of the nucleic acid [62]. When compared to the linear cyclodextrin-containing polycation (CDP), the imidazole-containing CDP (imCDP) displayed several mechanistic differences in its delivery of nucleic acids. Whereas imCDP bound nucleic acids (including oligonucleotides, siRNA, and plasmids) more tightly than CDP as demonstrated with cell-free assays, the imCDP released nucleic acids more readily intracellularly. These findings were consistent with other investigators that showed that protonation of the histidines within the acidic endosomes would aid in the release of nucleic acids. Besides the ability to lyse endosomes more readily, the imidazole-containing CDP polyplex had enhanced extracellular stability and greater transfection efficiency than CDP polyplexes [62].

Several groups have noted that histidines stabilized polyplexes to the disruptive effects of serum [6,7,12,32]. For example, the silencing effect from siRNA polyplexes remained constant in the presence of serum for 24 h with the histidine-containing polyplexes, whereas silencing was greatly reduced with the non-histidine polyplexes [12]. Moreover, poly-L-histidine was noted to form complexes with DNA in the presence of 2 M NaCl, indicating that non-ionic bonds were involved in the interactions [101]. Putnam and colleagues suggested that the stability of PEG-polyhistidine polyplexes may be due to hydrogen bonding [10]. By using isothermal titration calorimetry (ITC), Chou et al. demonstrated that histidines within the HK peptides formed non-ionic interactions with nucleic acids [12]. Whereas ITC showed an initial endothermic interaction of branched polylysine and siRNA, it demonstrated an exothermic interaction of the branched histidine-lysine peptide and siRNA. To investigate the type of non-ionic bond, NMR provided direct evidence that histidines formed hydrogen bonds with siRNA [12]. In this interaction, the imidazole forms hydrogen bonds with nucleic acids as a hydrogen donor through its δ1N-H tautomer, despite the ε2N-H tautomer being the most common tautomer in proteins [5].

**Figure 5:**
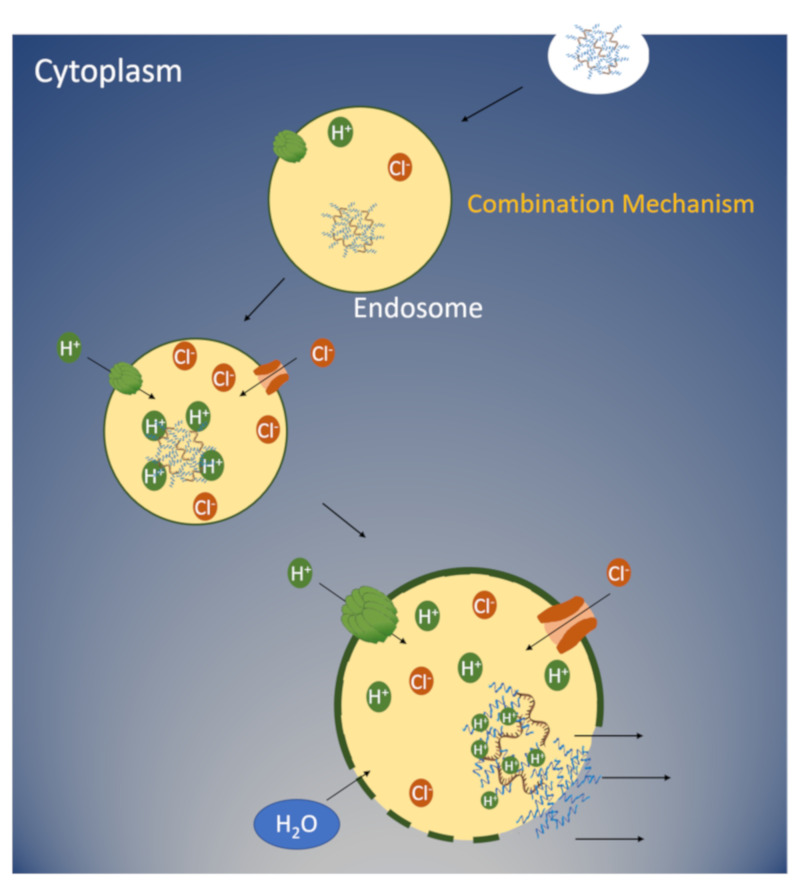
The combination endosomal lysis mechanism. Both the proton sponge mechanism with osmotic swelling and the interaction of the carrier with the endosomal membrane are responsible for the escape of the nucleic acid.

As discussed in the introduction, there have been two distinct mechanisms put forth that might explain the role of histidines/imidazoles in carriers for lysis of endosomes (Figure 2) (see recent review by Vermeulen et al. [102]). Alternatively, a third possibility is the combination of these two mechanisms (Figure 5). The proton-sponge mechanism leading to osmotic swelling and the interaction of the peptide with the membrane complement each other in the release of the nucleic acid from the endosomes. The mechanisms underlying the release of nucleic acids from endosomes are probably multifactorial [102], but at least in some cases, one mechanism may predominate over another.

**Figure 6:**
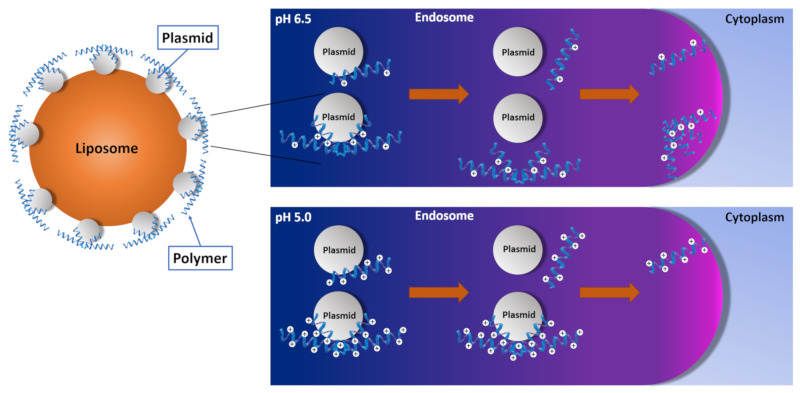
The optimal HK carrier (linear vs. branched) is dependent on the endocytic pH of cells. **Upper Panel**. At pH 6.5 in the endosomes of transformed and malignant cells, unpacking from the ternary complex (liposome, HK peptide, plasmid) occurs with the release of both linear and branched HK peptides from plasmid DNA. The branched HK peptide with the higher positive charge per molecule than the linear HK results in greater lysis and transfection. **Lower Panel**. At pH 5.0 in the endosomes of primary cell lines, unpacking occurs, but the branched peptide with an increased charge has a higher affinity toward DNA and is not released (or at least poorly released). In contrast, the highly protonated linear HK (with lower DNA affinity than the branched HK) is released from the plasmid DNA, enabling greater endosomal lysis and transfection.

For example, the unpacking of the H2K polyplex within the endosome with subsequent interaction of the peptide with the endosomal membrane appears to have the primary role in endosomal lysis [9]. Whereas branched HK peptides combined with liposomes demonstrated significantly higher transfection in malignant and transformed cells, the linear HK-liposome combinations were superior as carriers of plasmids in primary cells [9]. Moreover, there was a direct relationship between the degree of branching and transfection with HK peptide carriers in malignant cells. In contrast, there was an inverse relationship between the degree branching and the optimal HK peptide as a carrier of nucleic acids for primary cells. These results suggest binding and release of the HK peptides from the polyplexes were important in transfection by these carriers. Based on these findings, our group hypothesized that the pH the endo-lysosomal vesicles were lower in primary cell lines compared to transformed (or malignant) cells. Indeed, there was a strong association between the optimal type of HK polymer and the pH of endolysosomal vesicles [9]. There was also an association between the transfection findings of these lipopolyplexes and polyplexes. Although linear HK peptides as carriers by themselves were ineffective in the cells tested, the branched HK peptides were effective carriers of plasmids in malignant and transformed cells but not in primary cell lines. Thus, there was a correlation between the branched HK peptides and the branched HK-liposome combination to transfect cells. We think it is difficult for the proton-sponge hypothesis with endosomal swelling and lysis to adequately explain these findings. With the proton-sponge theory, it is not clear how the linear and branched polymers could be the optimal carriers in primary and transformed cells, respectively. The release of the HK peptides from the polyplexes with the subsequent interaction of peptides with the endosomal membranes offers a more straightforward explanation for these results (Figure 6).

Release and interaction of imidazole-containing carriers with the endosomal membrane may not always be the primary mechanism. It is difficult to understand how protonation of imidazoles on the polar-lipid heads of liposomes or micelles (as carriers of nucleic acids) would lead to the disruption or the release of the nucleic acid from the carrier. As Ripoll et al. suggest, the proton-sponge theory is likely the primary mechanism for enhanced siRNA silencing for their micelle [103]. We think that the number, location, and dispersion of histidines and imidazoles within the carrier are critical in what mechanism may be primary.

## 8. Challenges and Lingering Questions

Many of the challenges for histidine-rich peptide carriers are shared by other polymeric nucleic acid carriers. With few exceptions [104], the inability to characterize more fully the in vivo biophysical properties of polyplexes limits their successful development for the treatment of human diseases. These properties of the nanoparticles include their size, shape, and protein corona after systemic injection. This lack of characterization in vivo (vs. in vitro) may be particularly challenging for some polyplexes which may not be stable. Furthermore, even with demonstrative efficacy in the animal model (i.e., antitumor efficacy) with these polyplexes, there may be poor correlation between murine models and humans because of the lack of stability and the longer circulation time in humans. Pharmacokinetic and pharmacodynamic studies will not answer the question of how the shape and size of these polyplexes have been affected by their transit through the blood vessels to the target tissue. New strategies and methods are required to characterize the in vivo biophysical properties of histidine-rich polyplexes. For improved correlation between small and large animal models and for improved efficacy in humans, targeted and PEGylated polyplexes may need to be stabilized further via various methods such as modifying the peptides with lipids or using cross-linking approaches (i.e., oxidized cysteines) [49,80].

The inability to predict an immune response also hinders the development of histidine-rich carriers. Whether from reviewers about grant proposals or from companies, we are frequently asked about the innate and adaptive immune responses of the histidine-rich polymeric carriers. For histidine-rich polyplexes, the variability of the innate response differs markedly and may be dependent on the sequence of the histidine and lysine polymers [90,105]. Further studies identifying what histidine sequences induce or restrict the induction of cytokines are needed in developing carriers for vaccine and non-vaccine strategies.

Other than the LAH4 peptide or the histidinylated-polylysine polymer used as vaccine carriers for nucleic acids [51,90], little is known about the adaptive immune response to carriers discussed in this article. Whereas the algorithm software for MHC class I binding is very good in predicting CD8+T cell responses, the software for MHC class II binding and CD4+ response is less predictive but has improved in the last several years. The algorithms are not particularly predictive for antibody (B-cell) responses toward peptides, which include the histidine-rich polymers [106]. Nevertheless, based on steady advances in the last 10 years, these algorithms are anticipated to improve the predictability of B-cell responses to histidine-rich peptides. Our attempts to develop antibodies in mice and rabbits toward the branched HK models have been difficult. Although somewhat encouraging for our purposes, a lack of immune response in animals is far from predicting whether humans will have a similar response toward the histidine-lysine polymers [107]. Although predictive programs will no doubt improve, promising histidine-carriers at the present time may be thwarted by innate and immunological responses. These immune responses may not be discovered until primate studies or clinical trials have been initiated.

There are also specific questions about histidine-rich peptides raised by this review. For instance, why does a polyplex comprised of multiple linear clusters of histidine and lysines resist the disruptive effects of serum [37,38]? Other studies have demonstrated that intermingled sequences of histidines and lysines from linear or branched peptides are able to resist the effects of serum [31]. Although binding affinity, uptake, transfection, and descriptive studies of polyplexes have greatly improved these carriers, little is known about the specific atomic interactions between histidine-rich nanocarriers and nucleic acids. High-resolution methods such as X-ray crystallography to study the atomic interactions between peptides and nucleic acids are beyond the current grasp of scientists because of technical issues with crystallization. Other methods to define histidine-nucleic interactions include NMR and molecular dynamic simulations. As previously discussed, NMR showed that histidines/imidazoles formed hydrogen bonds with nucleic acids [12], and further studies should be able to define the specific functional groups on the nucleotide which form the hydrogen bonds. Alternatively, molecular dynamic simulations have been done to understand the interactions between polymers [108,109,110], including PEI and polylysine, with oligonucleotides. Similar studies could readily be applied to histidine-rich polymers and oligonucleotides.

Finally, complexed multilayered carriers will need to overcome barriers with production scale-up for commercial manufacturing. Even mundane issues that academicians rarely consider, such as long-term storage or lyophilization [111], may prevent a promising therapeutic approach from progressing into more advanced clinical trials. Before and after lyophilization, maintaining the biophysical properties and transfection of polyplexes has been problematic in some cases with our HK polymers. This was very much dependent on the HK polymer and nucleic acids. If the obstacles of stability, immune responses, and production can be surmounted, important concepts and insights from these challenges should emerge and be incorporated into future histidine-rich carriers.

## 9. Conclusions

Over the past 20 years, there have been significant advances in nucleic acid carriers modified by histidines or histidine-rich domains. We have described several properties of histidines, primarily emanating from their buffering of acidic endosomes. These roles from the protonated histidines in the enhancement of delivery of nucleic acids to their targets include osmotic swelling with lysis of endosomes, unpacking of the carrier complex, and release of the nucleic acids to enable the carrier to interact with the endosomal membrane.

Although these mechanisms for causing endosomal lysis are likely overlapping, one of the mechanisms may be predominant and be dependent on the location of the histidine-rich domains within the carrier. Besides its pH buffering properties, other properties of histidines include its ability to stabilize nanoparticles through hydrogen bonding and aromatic interactions. Numerous groups have demonstrated that histidine-lysine polyplexes were more stable with respect to the effects of serum than polylysine polyplexes. Nonetheless, these histidine-rich nanoparticles may require further stabilization such as PEGylation.

Another property of histidine is its ability to form coordination bonds with transitional metals [41]. One such study utilized the ability of zinc to bind with histidines, which provided a protective shield surrounding the nanoparticle [100]. Other studies have demonstrated that zinc added to histidine polyplexes increases transfection [28,39], perhaps by increasing the stability of the polyplexes. Although zinc could potentially be used as a cross-linker for histidine-rich polyplexes, the use of oxidized cysteines is probably a better choice. Numerous groups have determined that the judicious use of cysteines improves transfection [25,27,47,49], presumably by stabilizing the polyplex. We are not aware of any histidine-rich polymer in which cysteines have been added that transfection has not increased. Although oxidized cysteines on the surface of the nanoparticle may become reduced and re-oxidized with other cysteines, this is highly unlikely for PEGylated polyplexes.

For two decades, histidine-lysine polymers have been used to transport nucleic acids, but other cationic agents have also been substituted for lysines to bind nucleic acids. Direct comparison determined that the ornithine-histidine carrier was superior as a transfection carrier compared to the lysine-histidine carrier [45]. In addition, with oligo(ethanamino) amide branched polymers, cationic segments of diaminoethanes were substituted for lysines to bind the nucleic acids [48,49]. Besides the DNA binding component, the cationic segment of diaminoethanes also has unprotonated amino groups, which enable the buffering of endosomes. Because of the low molecular weight compared to lysines, the diaminoethane building blocks with a positive charge, buffering properties, and the potential to form hydrogen bonds may prove superior to lysines in histidine-rich polymers.

Instead of substituting cationic agents for lysines, another strategy has been to modify polyhistidine to enhance its solubility. Above pH 6.0, polyhistidine precipitates in aqueous solutions [70]. As a result, modifications of polyhistidine have been done by PEGylation [10], glycosylation [71], amination [72], methylation [73,74], and most recently, by carboxymethyl groups [76]. Thus far, the partial modification of polyhistidine with the negatively-charged carboxymethyl groups (CM-PLH) has been the only strategy used in vivo [76]. When systemically delivered, the ternary polyplex of PBAE, water-soluble CM-PLH, and luciferase plasmid resulted in a significantly higher luciferase expression in tumors than the binary PBAE-plasmid complex.

Most carriers are not effective for all forms of nucleic acids [112]. This was observed in some of the early studies of histidine-rich polymers in which low molecular weight reducible polymers were effective carriers of siRNA, whereas larger reducible polymers were effective carriers of plasmid DNA [26]. An alternative solution to varying the molecular weight is altering the sequence of histidines and lysines. HK polymers with similar molecular weight but with different sequences of histidine and lysine were effective carriers of plasmids or siRNA [31,86,105]. With mRNA, lipopeptides or liposomes are necessary to obtain effective transfection in vivo [113]. Thus far, histidinylated lipopolyplexes have proved to be effective carriers of mRNA in vivo. These lipopolyplexes vaccines have shown therapeutic antitumor activity in three pre-clinical tumor models [96].

In summary, we have reviewed the widespread use and progress of histidines containing nanocarriers to improve transfection. Recent strategies will likely facilitate the identification of improved histidine-rich carriers by testing libraries of polymers through an in vivo selection process [114,115]. To date, histidines or histidine-rich peptides have been incorporated into polymers, conjugated to lipids, phages, and mesoporous silica nanoparticles, as well as form shields around nanoparticles. Although the addition of histidines to diverse nanoparticles has improved their transfection, understanding the interactions of histidine-rich domains with nucleic acids and biological membranes will likely be necessary to realize the full potential of histidine-modified nanoparticles.

## Figures and Tables

**Table 1:** Representative histidine/imidazole-lysine carriers for nucleic acids.

Polymers	Nucleic Acid	In Vitro /In Vivo	Comments	References
Histidylated polylysine	pDNA ^1^	Y/N	Presence of serum (20%) had little effect on transfection; Increased DNA transfection more than 4-logs compared to polylysine (DP-190)	[6]
Imidazole-containing polylysine	pDNA	Y/N	Polylysine in which 86.5% of ε amines were modified by imidazoles had similar transfection efficiency as PEI but less toxicity	[23]
Histidylated oligolysine	ODN	Y/N	Low MW carrier (DP-19) more effective than HMW (DP-190); 80% reduction of ICAM-1 expression with peptide-ODN compared to peptide-scrambled ODN polyplexes in vitro	[24]
CH_6_K_3_H_6_C	pDNA/ siRNA	Y/N	Varying MW of reducible polymer were made; size dependent efficacy for carrier of siRNA and pDNA; addition of targeting malaria ligand increased efficacy to hepatocytes in vitro	[25]
CH_6_K_3_H_6_C	pDNA/ mRNA	Y/N	HMW reducible polycation carrier was effective for mRNA and plasmids in vitro	[26]
CHK_6_HC	pDNA	Y/N	Histidine-containing peptide enhanced gene expression by up to 7-fold compared to CK_8_C peptides; N- and C-terminal cysteines oxidized to form stable cross-linked polyplexes.	[27]
Histidylated polylysine	pDNA	Y/N	Zinc chloride added to polymer (DP-190) increased transfection efficiency significantly	[28]
cRGD-hK	pDNA	Y/Y	cRGD-hK (CRGDCF-(K[H-]KKK)_6_) polyplexes injected iv enhanced DNA transfection efficiency in tumor xenografts compared to normal tissues	[29]
KGH6	pDNA	Y/Y	KGH6, a 6th generation dendrimer, in which the terminal amino acids were histidines, showed a 3-logs higher transfection efficiency in cells when polyplex formed at pH 5.0 than 7.4	[30]
H2K4b-T	pDNA	Y/N	H2K4b-T enhanced plasmid transfection efficiency compared to H2K4b	[31]
H3K8b	siRNA	Y/N	An effective eight-branched carrier for siRNA with low toxicity	[32]
PEG-CH_12_K_18_	pDNA	Y/Y	An effective triblock carrier for plasmids expressing luciferase in vitro and in vivo in lung models; worm-like structure of polyplex	[33]
PEG-CH_12_K_18_	pDNA	Y/Y	Enhanced silencing of luciferase with intratumoral injection in a neuroblastoma model.	[34]
H2K	pDNA	Y/Y	In contrast to the H2K4b, the linear H2K carrier had a low transfection in vitro, but a high transfection of tumors in vivo. Transcytosis was mediated by NRP-1 receptor	[35,36]
(KHKHKHKHKK)_6_-FGF2	pDNA	Y/N	High transfection efficiency of FGF-2 targeted polyplex but not in the presence of serum	[37]
(KKKHHHHKKK)_6_-FGF	pDNA	Y/N	Clustered lysines and histidines improved stability and transfection compared to non-clustering of carrier, particularly in serum	[38]

^1^ pDNA, plasmid; Y or N, yes or no for whether in vitro and in vivo studies were performed; DP, degree of polymerization; ODN, oligodeoxynucleotide; MW, molecular weight; LMW, low molecular weight; HMW, high MW; CHK_6_HC, CHKKKKKKHC, one letter amino acid code, subscript represents the number of repeats for amino acid or sequence; cRGD, cyclic RGD; NRP-1, neuropilin-1, FGF-2, fibroblast growth factor-2.

**Table 2:** Sequence-specific histidine carriers: supplements and alternatives to lysines.

Polymer	Nucleic Acid	In Vitro/ In Vivo	Comment	Reference
LAH4	pDNA ^1^	Y/N	LAH4 and PEI polymers have comparable transfection efficiency in several cell lines; number and positions of histidines in peptide were important	[43]
	siRNA	Y/N	LAH4 and derivatives have improved siRNA transfection compared to cationic liposomes and PEI in a retinoblast cell line.	[44]
O_10_H_6_	pDNA	Y/Y	O_10_H_6_, a 16-mer, has a higher DNA transfection efficiency in dendritic cells than K_10_H_6_ with lower toxicity; O_10_H_6_ polyplexes elicited antigen-specific INFγ in vivo	[45]
	ODN	Y/ N	Microspheres coated with O_10_H_6_ polyplexes markedly increased accumulation of ODN in dendritic cells than O_10_H_6_ polyplexes alone.	[46]
C-H_5_-TAT-H_5_-C (CH_5_RK_2_R_2_QR_4_H_5_C)	pDNA	Y/Y	Of modified TAT peptides, the C-H_5_-TAT-H_5_-C carrier improved gene transfection the most in vitro. PEI-25kD and the modified TAT carriers gave similar gene expression after intrathecal injection.	[47]
Oligo(ethana-mino) amide branched polymers (Gtt, Stp, Sph)	pDNA	Y/Y	Enhanced pDNA transfection efficiency both in vitro and in vivo. Gtt, Stp, and Sph have buffering properties and positive charges (replacing lysines). Multimers of Gtt-Histidine, Stp-Histidine, and Sph-histidines were incorporated in linear and branched polymers.	[48]
	pDNA	Y/Y	Ligand targeted Polyplexes toward cMet enhanced receptor-specific gene transfer with intratumoral and intravenous injections. PEGylated targeted and non-PEGylated polymers were mixed together with pDNA to form an effective and stable polyplex for studies in vivo.	[49]

^1^ pDNA-plasmid; PEI, polyethylenimine; ODN-oligodeoxynucleotide; Gtt, Glutaroyl-triethylene tetramine; Stp, succinoyl tetraethylene pentaamine; Sph- succinoyl pentaethylene hexamine.

**Table 3:** Histidine/imidazoles conjugated to non-biodegradable and biodegradable polymers.

Polymer	Nucleic Acid	In Vitro /In Vivo	Comment	Reference
**NON-BIODEGRADABLE**				
Histidinylated linear PEI (H-lPEI)	pDNA	Y/N	High transfection efficiency with lower toxicity than linear PEI (IPEI) in vitro	[55]
H-lPEI/IPEI	pDNA	Y/N	H-lPEI/IPEI with 57 and 67% lPE had similar transfection but lower toxicity than IPEI.	[56]
PAMAM-G4-H3-R	pDNA	Y/N	Histidine-arginine peptide conjugated to polyamidoamine dendrimer had higher transfection and lower toxicity than PEI in various cell lines	[57]
**BIODEGRADABLE**				
Urocanic acid (UA)-modified chitosan	pDNA	Y/N	DNA transfection efficiency in 293T cells was enhanced with the addition of UA to chitosan.	[58]
	pDNA	N/Y	UA chitosan in complex with plasmid expressing PTEN via aerosol reduced number of lung tumors in a K-ras mouse model	[59]
H_6_R_6_-modified chitosan (CS)	siRNA	Y/Y	Modified CS has higher transfection efficiency and improved endosomal escape. Modified CS carrier of survivin siRNA reduced breast cancer growth in vivo.	[60]
Histidine-cysteine modified trimethyl CS	pDNA	Y/Y	Histidine-modified CS polyplexes modestly increased transfection compared to unmodified CS polyplexes but histidine-polyplexes had lower transfection than arginine-modified CS polyplexes.	[61]
Imidazole-modified cyclodextrin (imCDP)	pDNA	Y/N	imCDP confers increased binding to DNA, enhanced released in acidic environments, increased buffering capacity and transfection efficiency in vitro.	[62]
Imidazole modified-curdlan	pDNA/ siRNA	Y/N	Enhanced endosomal escape and efficiently delivered plasmid and siRNA into cancer cells	[63]
Poly(imidazole-DMAEA) phosphazene (PIDP)	pDNA	Y/N	DNA transfection in 293T, COS-7 and Hela cells was higher with PIDP than the polymer control and PEI-25 kD, and had lower cytotoxicity.	[64]
End-Capping of modified PBAE	pDNA	Y/Y	End-capping of the PBAE polymer, C32, with histidine showed reduced transfection compared to end-capping with primary and tertiary amines.	[65]
siRNA-aptamer chimeras with polyhistidine	siRNA	Y/N	A platform for siRNA with a targeting aptamer, a dsRNA binding domain and polyhistidine for endosomal escape. Polyhistidine domain with 18 histidines more effective than 6 histidines.	[66]

PEI, polyethyleneimine; pDNA, plasmid; PAMAM-G4, generation 4 polyamidoamine dendrimer; UA, urocanic acid; CS, chitosan; siRNA, short interfering RNA; imCDP, imidazole-modified cyclodextrin; DMAEA, dimethylaminoethylamino; PIDP, poly(imidazole/DMAEA) phosphazene; PBAE, poly(β-amino esters); dsRNA, double-stranded DNA binding domain.

**Table 4:** Modification of polyhistidines to enhance solubility and the capacity to import nucleic acids.

Polymer	Nucleic Acid	In Vitro /In Vivo	Comment	References
PEG ^1^-polyhistidine	pDNA	Y/N	Both linear and comb-shaped PEG-polyhistidines conjugates were synthesized. Some of the formulations were relatively stable in size for 7 days at neutral pH.	[10]
Glycosylated-polyhisti- dine/transferrin-targeted polylysine	pDNA	Y/N	Ternary polyplex significantly more effective than the binary transferrin-polylysine polyplex	[71]
Aminated polyhistidine	pDNA	Y/N	Enhanced membrane disruptive ability at lower pH and modest increase in transfection compared to polylysine	[72]
Methylated Polyhistidine	siRNA	Y/N	Low percentage of methylation (25% vs. 68%, 87%) of PLH-Me was most efficient for gene silencing and gene expression	[73,74]
Carboxymethyl polyhistidine/PEI	pDNA	Y/N	The ternary polyplex (CM-PLH, PEI, plasmid) enhanced DNA transfection efficiency 300 times higher than the PEI binary polyplex	[75]
CM-PLH/ poly (β-amino ester)	pDNA	Y/Y	Ternary polyplex (CM-PLH, PBAE, plasmid) had 4-fold higher gene expression than non-coated PBAE polyplex in tumors in vivo	[76]

^1^ PEG-polyethylene glycol; PLH-Me, Methylated Poly(L-histidine); siRNA, short-interfering RNA; CM-PLH, carboxymethylated-polyhistidine; PBAE, poly (β amino ester).

**Table 6:** Pre-formed nanoparticles modified with histidines and imidazoles.

Carrier	Nucleic Acid	In Vitro /In Vivo	Comment	Reference
Fusogenic H5WYG peptide conjugated to virus-like particles	siRNA	Y/N	About seventy-five “H5WYG” peptides per particle. Without fusogenic peptide, the particles containing siRNA were significantly less effective targeting cyclins in Hep3B cells	[22]
Mesoporous silica nanoparticles with L-histidine (MSN-His ^1^)	pDNA	Y/Y	Improved pDNA transfection efficiency both in vitro and in Achilles tendons in vivo compared to unmodified MS	[98]
Imidazole linked to dendritic mesoporous silica nanoparticle	pDNA/Doxoru-bicin	Y/Y	Carrier exhibited high drug loading capacity, pH-sensitive targeting and drug release; marked tumor inhibition with doxorubicin and shSurvivin in vivo	[99]
Imidazole framework film covering MSN	siRNA/Doxoru-bicin	Y/N	Ultrathin zinc-imidazole film (or ZIF-8) on MSN adsorbed siRNAs with high efficiency and released siRNA and small drugs readily inside the cells	[100]

^1^ MSN-His, mesoporous silica nanoparticles modified by histidines; ZIF-8, zeolithic imidazole framework.

## Abbreviations

DEAE-dextran	diethylaminoethyl-dextran
PAMAM	polyamidoamine
PEI	polyethylenimine
H5WYG	GLFHAIAHFIHGGWHGLIHGWYG (one letter amino acid code)
pDNA	plasmid
Y or N	yes or no for whether in vitro or in vivo studies were done
DP	degree of polymerization
ODN	oligodeoxynucleotide
HMW	high molecular weight
LMW	low molecular weight
CHK_6_HC	HMW reducible peptide containing multiple CHKKKKKKHC repeats
CH_6_K_3_H_6_C	HMW reducible peptide containing multiple CHHHHHHKKKHHHHHHC repeats
GFP	green fluorescent protein
cRGD	cyclic RGD ligand with oxidized cysteines that has the sequence, CRGDCF
HK	Histidine-Lysine peptide
PEG	polyethylene-glycol
PEG-CH_12_K_18_	PEGylatated copolymer made up of 12 histidines and 18 lysines
PEG-CK_30_	PEGylated polymer made up of 30 lysines
shRNA	Plasmid expressing short hairpin RNAi targeting luciferase
cRGD-hK	cyclic RGD conjugated to N-terminal of polylysine in which every 4th lysine modified by a histidine
KGH6	6th generation lysine dendrimer with terminal ends modified by histidines
H2K4b-T	a 4-branched peptide with a predominant sequence of -HHK- and a histidine-rich domain off the C-terminal end of the 3-lysine core (i.e., Tail)
H3K8b	8-branched peptide with a predominate sequence of -HHHK
H2K4b, H2K	a 4-branched and linear HK peptide with a predominant sequence of -HHK-
FGF-2	fibroblast growth factor-2
dKH	diffuse distribution of KH sequences with 6 multiple repeats
cKH	cluster KH—6 multiple repeats of clustered lysines and histidines
LAH4	peptide with sequence of KKALLALALHHLAHLALHLALALKKA
O_6_H_6_	blocked copolymer containing six ornithines and histidines
K_16_	peptide with 16 lysines
DC	dendritic cells
TAT	cell-penetrating peptide with sequence of RKKRRQRRRR
C-H_5_-TAT-H_5_-C	reducible peptide with sequence of CHHHHHRKKRRQRRRRHHHHHC
Gtt	glutaroyl-triethylene tetramine
Stp	succinoyl tetraethylene pentamine
Sph	succinoyl pentaethylene hexamine
cMET	tyrosine kinase receptor
siEG5-KLK	conjugate of siRNA targeting EG5 and apoptotic peptide, KLK
PAMAM-His3-Arg	polyamidoamine dendrimer with terminal ends modified by -HHHR
PAMAM-His2-Arg	polyamidoamine dendrimer with terminal ends modified by -HHR
PAMAM-His3-Arg	polyamidoamine dendrimer with terminal ends modified by -HR
PAMAM-Arg	polyamidoamine dendrimer with terminal ends modified by -R
lPEI	Linear PEI
H-lPEI	histidinylated linear PEI
UA	Urocanic acid
PTEN	Phosphatase and tensin homolog protein
Cs	Chitosan
R_6_H_6_-Cs	Chitosan modified with a blocked co-peptide of RRRRRRHHHHHH
CDP	cyclodextrin
imCDP	cyclodextrin modified with imidazoles
DMAEA	2-dimethylaminoethylamino
PIDP	polyphosphazene polymer modified with imidazoles and DMAEA
PDAP	polyphosphazene polymer modified with DMAEA
PBAE	poly(β-amino esters
PLH-Me	Polyhistidine containing methylhistidines, dimethylhistidine, and unmodified
CM-PLH	carboxymethylated-polyhistidine histidines
KALA	a cationic amphipathic *peptide*
H6-20	lipopeptide containing six histidines (also named NickFect70)
siGADPH	siRNA targeting GADPH
NRP-1	neuropilin-1
Chol	cholesterol
PD-1	program cell death protein 1
LPD	ternary complex (lipopolyplex) made up liposomes, polymer, and DNA
LPR	ternary complex (lipopolyplex) made up liposomes, polymer, and RNA
hK-liposomes	hK peptide conjugated to liposomes
His-Lip	Histidine-modified liposome
MART-1	Melanoma Antigen Recognized by T cells 1
LAMP1	lysosomal-associated membrane protein 1
MART-1-LAMP1	Fusion of the MART1 and LAMP1 proteins
MAN-LPR	Mannosylated targeted RNA lipopolyplex
Im-Hist-Lip	Imidazole-histamine modified liposomes
PEG-HpK	PEG-histidylated-polylysine
Tri-man	tri-antenna of α-D-mannopyranoside
Tri-Man-Im-Hist-Lip	Tri-Man ligand coupled to Im-Hist-liposome
DOTAP	1,2-dioleoyl-3-trimethylammonium-propane
IPEI	linear polyethylenimine containing 16% histidines
MSN	mesoporous silica nanoparticles
MSN-NH2	mesoporous silica nanoparticles with aminopropyltriethoxysilane
MSN-His	mesoporous silica nanoparticles with aminopropyltriethoxysilane and histidines
SL-IDMSN	MSN modified by imidazoles
ZIF-8	zinc imidazole framework
VLP	virus-like particles
